# Application of Luteolin in Neoplasms and Nonneoplastic Diseases

**DOI:** 10.3390/ijms242115995

**Published:** 2023-11-06

**Authors:** Katarzyna Rakoczy, Justyna Kaczor, Adam Sołtyk, Natalia Szymańska, Jakub Stecko, Jakub Sleziak, Julita Kulbacka, Dagmara Baczyńska

**Affiliations:** 1 Faculty of Medicine, Wroclaw Medical University, 50-367 Wroclaw, Poland; katarzyna.rakoczy@student.umw.edu.pl (K.R.); justyna.kaczor@student.umw.edu.pl (J.K.); adam.soltyk@student.umw.edu.pl (A.S.); natalia.szymanska@student.umw.edu.pl (N.S.); jakub.stecko@student.umw.edu.pl (J.S.); jakub.sleziak@student.umw.edu.pl (J.S.); 2 Department of Molecular and Cellular Biology, Faculty of Pharmacy, Wroclaw Medical University, 50-556 Wroclaw, Poland; 3 Department of Immunology, State Research Institute Centre for Innovative Medicine, Santariškių 5, 08410 Vilnius, Lithuania

**Keywords:** luteolin, flavonoids, carcinogenesis, COVID-19, diabetes mellitus, skin diseases

## Abstract

Researchers are amazed at the multitude of biological effects of 3′,4′,5,7-tetrahydroxyflavone, more commonly known as luteolin, as it simultaneously has antioxidant and pro-oxidant, as well as antimicrobial, anti-inflammatory, and cancer-preventive, properties. The anticancer properties of luteolin constitute a mosaic of pathways due to which this flavonoid influences cancer cells. Not only is it able to induce apoptosis and inhibit cancer cell proliferation, but it also suppresses angiogenesis and metastasis. Moreover, luteolin succeeds in cancer cell sensitization to therapeutically induced cytotoxicity. Nevertheless, apart from its promising role in chemoprevention, luteolin exhibits numerous potential utilizations in patients with conditions other than neoplasms, which include inflammatory skin diseases, diabetes mellitus, and COVID-19. This review aims to present the multidimensionality of the luteolin’s impact on both neoplastic and nonneoplastic diseases. When it comes to neoplasms, we intend to describe the complexity of the molecular mechanisms that underlay luteolin’s anticancer effectiveness, as well as to prove the usefulness of integrating this flavonoid in cancer therapy via the analysis of recent research on breast, colon, and lung cancer. Regarding nonneoplastic diseases, this review aims to emphasize the importance of researching the potential of luteolin in areas such as diabetology, virology, and dermatology as it summarizes the most important discoveries in those fields regarding its application.

## 1. Introduction

**Figure 1:**
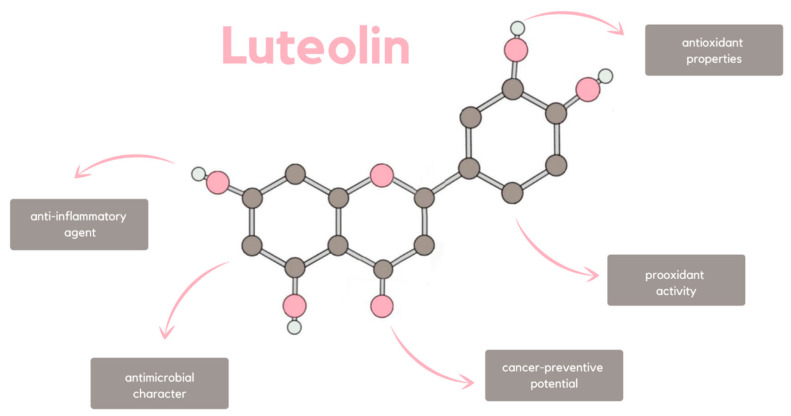
Visualization of the chemical structure of luteolin and its main properties [7,8,9,10].

The diversity of nature-derived substances that influence human organisms does not cease to astonish the world of medicine with its limitless potential. Among the cornucopia of their possible applications, 3′,4′,5,7-tetrahydroxyflavone, more commonly known as luteolin, distinguishes itself by the multidimensionality of its biological effects (Figure 1). It constitutes a component of flavonoids, polyphenols commonly found in plants that were proven beneficial for human health in specific concentrations [1]. Naturally, luteolin can be found in numerous vegetables, such as raw spinach, dried parsley, and cabbage, as well as fruits, plants, and herbs [2,3,4]. Some of them have been used as traditional Chinese medicine for inflammatory diseases and hypertension [5]. Thus, it can be regarded as a common nutrient, which simultaneously has antioxidant and pro-oxidant, as well as antimicrobial, anti-inflammatory, and cancer-preventive, properties [6,7] (Figure 1). The enumerated pharmacological activities are functionally connected to each other, as cancer-preventive function is strongly correlated with the anti-inflammatory potential. Nevertheless, the anticancer properties of luteolin constitute a mosaic of pathways due to which the described flavonoid affects cancer cells. Not only is it able to induce apoptosis and inhibit cancer cell proliferation, but it also suppresses angiogenesis and metastasis. Moreover, the described flavonoid does succeed in cancer cell sensitization to therapeutically induced cytotoxicity.

**Figure 2:**
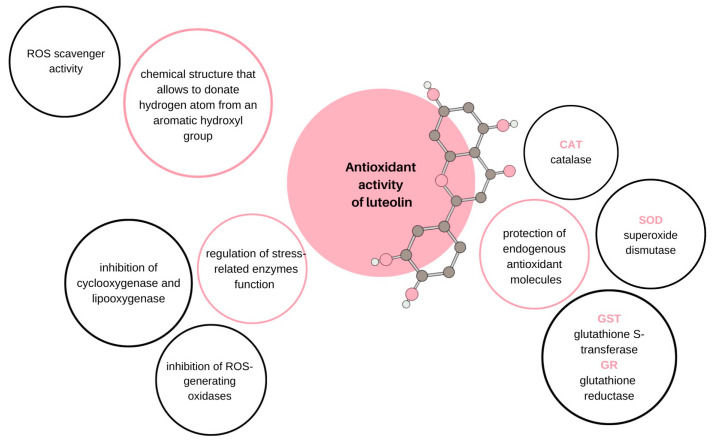
Schematic visualization of connections between foundations of luteolin’s antioxidant properties [6,11,20,21].

In plants, luteolin usually occurs in its glycosylated form, hydrolyzed during absorption releasing free luteolin [8]. Then, during intestinal passage, some of the luteolin molecules are methylated or transformed into glucuronides [11]. Methylation can lead to chrysoeriol and diosmetin synthesis, catalyzed by catechol-O-methyltransferase in rats [12]. Orally administered luteolin is known to be delivered to circulation mainly in the form of conjugated metabolites; however, comprehensive data regarding luteolin’s pharmacokinetic characteristics, tissue distribution, and the excretion pathway of each metabolites remain unavailable. Importantly, luteolin can be applied in central nervous system disease therapy, as it may cross the blood–brain barrier [13]. Nevertheless, pharmacokinetic research suggests that, at least in rats, the described flavonoid is effectively absorbed but then extensively metabolized, which determines the low bioavailability of luteolin, ranging up to 17.5% [14]. Importantly, even though a few years ago the heat stability of luteolin was taken for granted [15], thermal treatment significantly diminishes the cancer-preventive properties of luteolin, influencing its cytotoxic activity as well as its ability to inhibit cellular migration and endothelial cell angiogenesis. Thus, the antitumor potential of native luteolin is more remarkable than the potential of heat-treated luteolin [16]. Regarding its properties, luteolin, like other flavonoids, is an antioxidative agent that inhibits ROS-induced DNA, lipid, and protein damage [17]. Examples of ROS include hydrogen peroxide (H_2_O_2_), singlet oxygen (^1^O_2_), and hydroxyl radical (OH-), which exhibit high reactivity to numerous biological targets. However, their role is not limited to intracellular devastation, as they act as signaling molecules that regulate physiological processes. Moreover, those molecules are significant for neuromodulation, immunomodulation, ion transport, and apoptosis [18]. Thus, the presence of ROS themselves is not an entirely negative phenomenon, in contrast to their sudden excess, which causes oxidative stress. The antioxidant activity of luteolin is founded upon more than one molecular mechanism, and the described complexity ensures its infallibility. Luteolin influences the function of many oxidative stress-related enzymes. These include ROS-generating oxidases, such as xanthine oxidase, the activity of which is effectively suppressed by luteolin [19]. Luteolin also inhibits enzymes catalyzing cellular component oxidation, such as cyclooxygenase and lipoxygenase [20]. Additionally, the described flavonoid can protect and augment endogenous antioxidant molecules, for instance, superoxide dismutase (SOD), catalase (CAT), as well as glutathione-S-transferase (GST) and glutathione reductase (GR) [21]. Moreover, luteolin can be oxidized, playing the role of an ROS scavenger. Its chemical structure shared across flavonoids allows it to donate hydrogen atoms from an aromatic hydroxyl group [22]. The combination of the described mechanisms that allow luteolin to be regarded as an antioxidant emphasizes the multidirectionality of its influence that appears useful in terms of its possible clinical application (Figure 2).

**Figure 3:**
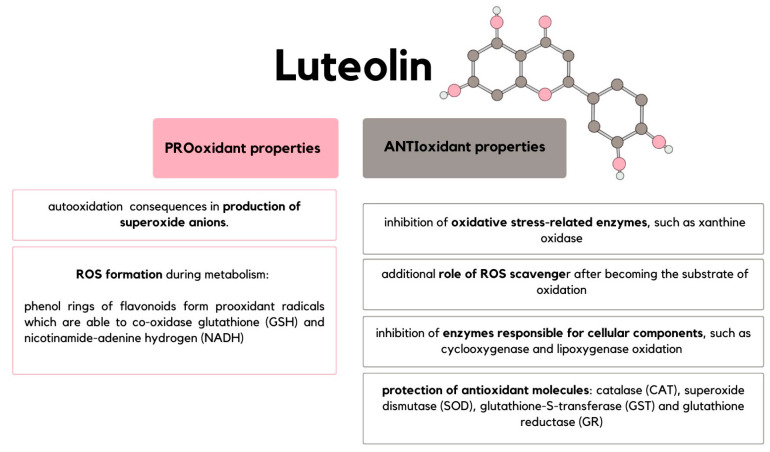
Graphical summary of antioxidant and pro-oxidant properties of luteolin [6,11,20,21,22,23,24,25,26].

Unexpectedly, despite the proven antioxidant potential of flavonoids, there is more and more evidence suggesting that they also exhibit pro-oxidant properties (Figure 3) [23]. Flavonoids can undergo autooxidation that is catalyzed by transition metals and results in the production of superoxide anions. There are also studies suggesting that during the metabolism of flavonoids, their phenol rings form pro-oxidant radicals that can co-oxidase glutathione (GSH) as well as nicotinamide-adenine hydrogen (NADH), which is connected with ROS formation [24]. The dualism of luteolin-induced redox status makes the described flavonoid reveal one of its contradictory properties depending on differentiated factors. They are its concentration, the source of free radicals, and the characteristics of the cell microenvironment [23]. For instance, as flavonoids have chelating properties, their antioxidant activity is determined by the concentration of copper (Cu), cadmium (Cd), and iron (Fe) in cells. It has been proven that under low Fe^3+^ concentrations, luteolin acts as an antioxidant agent, whereas increased concentration stimulates its pro-oxidant activity [25,26]. The understanding of the mechanisms by which luteolin can behave in certain ways is incomplete and thus requires further examination. If the determination of the luteolin activity in certain microenvironments was predictable, the potential of its clinical application could be significant. In the role of an antioxidant, luteolin might protect cells from oxidative stress and, as oxidative stress is closely associated with mutagenesis, in this way, prevents neoplastic transformation [27]. Contrarily, its pro-oxidant activity could underlay its ability to induce the apoptosis of the tumor cells [28].

Luteolin has been proven to act as an anticancer agent in lung, prostate, colon, and pancreatic cancer and glioblastoma [29,30,31]. It also suppresses cancer development in vitro and inhibits tumor cell proliferation in vivo. Moreover, the described flavonoid can activate the cell cycle arrest and induce apoptosis. The connection between luteolin and neoplastic diseases is complex and is discussed more specifically in this paper. Nonetheless, apart from anticancer and cancer-preventive properties, 3′,4′,5,7-tetrahydroxyflavone exhibits the ability to modulate inflammatory processes, as it can regulate differentiated signaling pathways, such as the TLS signaling pathway and NF-κB, as well as suppress proinflammatory mediators, among other things IL-6, IL-8, IL-17, and IL-22 release, and thus has the potential to improve therapies in many medical fields [32]. Apart from the dermatological and diabetological fields described in this review, one of the newest articles about luteolin presented its analgesic properties [33]. It is known that luteolin can act as an important anti-inflammatory factor; it may also be used in pain management—especially in chronic conditions and neuropathic pain [33].

Importantly, in the most recent research, luteolin has been proven to suppress the matrix stiffness-induced biological effects and the CXCR4-mediated yes-associated protein (YAP) signaling pathway in hepatocellular carcinoma (HCC) cells. An increase in tumor stiffness can be detected during the progression of the tumor due to increased ECM deposition. Intracellular tension, determined by the dynamic state of balance between the elastic resistance of ECM and contractile cytoskeleton, is the basis of the mechanism of sensing cellular stiffness. Exerting contractile force and sensing counter tension allow the mechano-cellular system to respond to pathological changes in ECM stiffness. In the intracellular world, matrix stiffness signals control mechano-sensitive activities through ubiquitin domain-containing protein 1 (UBTD1)-mediated YAP signaling pathway affected by CXCR4. Additionally, CXCR4 plays the role of a molecular switch, transducing a mechano-signaling pathway from matrix stiffness to the nucleus. Describing the process more carefully, through CXCR4, the levels of UBTD1 are decreased, leading to the degradation of YAP, an important mechano-transduction protein [34]. 

Although the therapeutic properties of luteolin seem promising, its potential toxicity and health impact must be taken into consideration [35,36]. There are presumptions that flavonoids may interact with other medications when co-administered and alter their pharmacotherapeutic profile [36]. As these substances may cross a placenta, toxicity can affect the unborn fetus [37]. On the other hand, some authors point out that flavonoids demonstrate mild cytotoxicity in normal, noncancerous cells only when they achieve high concentrations [36]. 

Currently, luteolin has been successfully brought to the market as a dietary supplement and is also incorporated into cosmetic products, as demonstrated by its nontoxic side effects. This safety is supported by data showing that the oral median lethal dose (LD50) in mice and rats exceeded 2500 and 5000 mg/kg, respectively, roughly translating to 219.8−793.7 mg/kg in humans. In animal and cellular investigations, luteolin demonstrated a lack of detrimental effects on healthy cells and did not result in noteworthy side effects, even at a relatively high concentration of 30 μM [38,39]. Research intended to examine luteolin’s ability to reverse changes caused by exposure to lead in rats showed no side effects at a dose of 50 mg/kg, oral, daily; thus, the researchers concluded that luteolin could possibly be administered as a dietary supplement in humans [40]. In research designed to examine the effectiveness and tolerability in white children with autism spectrum disorder of a dietary supplement containing two flavonoids (>95% pure), including luteolin (100 mg per 10 kg weight per day), for 26 weeks found transient irritability in subjects as the only side effect of treatment and, interestingly, a significant improvement in adaptive functioning as measured by using the Vineland Adaptive Behavior Scales age-equivalent scores [41]. 

In research aiming to find the steroid hormone activity of flavonoids, luteolin at a 10^−5^ M concentration showed the strongest estrogenic activity of all flavonoids [42]. However, another study showed the antiestrogenic effects of luteolin at a concentration of 10 mM and its ability to suppress the induction of the proliferation-stimulating activity of environmental estrogens [43]. This substance’s relatively low estrogenic potency when interacting with ERs could explain the seemingly contradictory outcome. Flavonoids can bind to and stimulate ERs when estrogen levels are insufficient. Nevertheless, owing to their comparatively modest estrogenic strength, which is 103 to 105 times less potent than 17-β-estradiol or luteolin, they may act as antiestrogenic agents by competing with estrogens for binding to ERs [38]. This luteolin characteristic may be desirable or undesirable depending on the clinical context. An important impact of luteolin on progesterone, estrogen, and glucocorticoid signaling has been noted in research which found out that at a concentration achievable by dietary supplementation of 8 μM luteolin achieves progestin antagonist activity that successfully inhibited the growth of luminal A subtype breast cancer cells. However, this beneficial feature was countered by luteolin’s estrogen agonist activity, which stimulates growth in an endometrial cancer model in a similar dose range. Thus, because of its ability to inhibit the protective effect of progestins via progestin antagonist activity, supplementation with luteolin should be contraindicated for women at risk of endometrial cancer [44]. In research aiming to examine the luteolin’s impact on experimental colitis models, NF-κB^EGFP^ transgenic mice were given a diet containing 2% luteolin, and acute colitis was induced using 3% dextran sodium sulfate (DSS). In addition, the development of spontaneous colitis was assessed in IL-10^−/−^NF-κ^BEGFP^ transgenic mice fed a diet with 2% luteolin [45]. Interestingly, the NF-κB^EGFP^ transgenic mice exposed to luteolin exhibited more severe DSS-induced colitis, as evidenced by weight loss and histological scores, when compared to the mice on a control diet. However, spontaneous colitis in the IL-10^−/−^NF-κB^EGFP^ mice was notably mitigated when they were fed a diet containing 2% luteolin. The worsening of colitis in luteolin-treated mice exposed to DSS may be linked to an increased susceptibility of enterocytes to apoptosis induced by signals and a reduced capacity to initiate tissue repair [45]. These findings call for further investigations into the effects of luteolin on the development of colonic dysplasia and cancer and its role in chronic intestinal inflammation.

## 2. Carcinogenesis

**Figure 4:**
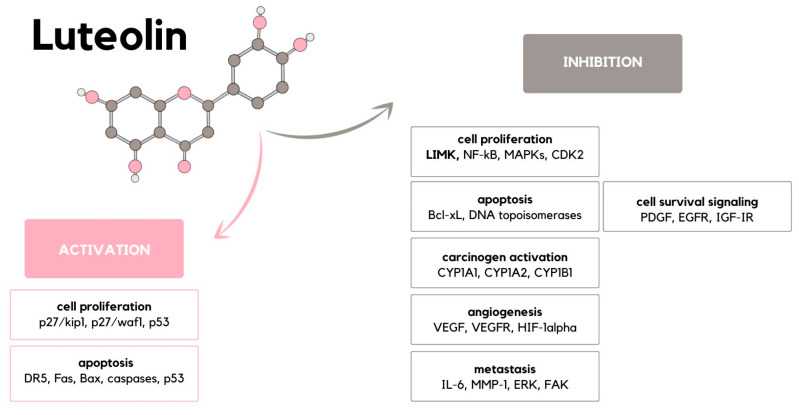
Schematic visualization of molecules that form specific cellular signaling pathways and the way they are influenced by luteolin [47,48,49,50,51,52,53].

There are numerous and complex ways in which luteolin can interfere with carcinogenesis, constituting a multistage process that leads to clonal expansion of a cell affected by carcinogenic mutation. In order to comprehend the multiplicity of the discussed flavonoid’s influence on neoplasia formation, brief characteristic of carcinogenesis stages is inevitable. Initiation, the first stage, occurs when promutagen becomes a mutagen due to P450 and other enzymes. Newly formed mutagen induces DNA alterations, including deletions, transitions, and transversions. Promotion, the second stage, occurs when those alterations favor cell growth and proliferation, which defines the irreversible tumorigenic character of changes. The cancer cell is then characterized by uncontrolled proliferative activity, the ability to invade surrounding tissues and evade immunological response, karyotypic instability, and the capacity to trigger angiogenic response [46]. The enumerated properties of neoplastic cells find their reflection in the alterations in cellular signaling pathways, with which luteolin can interfere via numerous molecular targets, presented in Figure 4. The most significant of those pathways are described below.

## 3. Cell Proliferation

**Figure 5:**
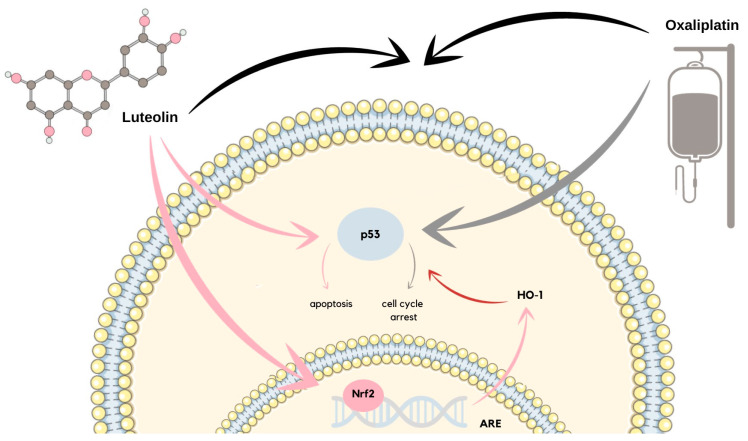
Schematic visualization of oxaliplatin and luteolin simultaneous administration effects [56,58].

Luteolin can activate and inhibit cell proliferation in various ways by inhibiting cell cycle progression or suppressing growth factor receptor-mediated cell proliferation signaling. Regarding the cell cycle, flavonoids inhibit tumorigenesis by arresting the cell cycle progression in specific checkpoints, which are G1/S and G2/M [54]. Luteolin can arrest the cell cycle during the G1 phase via different pathways, which was proven in, among others, lung cancer, gastric cancer, neuroblastoma, and colorectal carcinoma cells [55,56]. In one of the studies, the ability of luteolin to inhibit the LIMK kinase activity in lung cancer cell lines NCI-H1975 and NCI-H1650 was confirmed. The suspicion that LIMK is the major target of luteolin became a fact as after the LIMK expression knockdown, the flavonoid did not inhibit the tumor growth. As was validated, the described study also proved that luteolin application causes the arrest of the cell cycle during the G1 phase and induces apoptosis in a dose-dependent manner. Luteolin treatment regulates the expression of markers such as Bax, cleaved caspase-3, and cleaved caspase-7 [55]. In another study, growth inhibition of SH-SY5Y cells was investigated in a dose-dependent and time-dependent manner, which revealed that luteolin induced the G0/G1 cell cycle arrest. Moreover, the authors of the study discussed the flavonoid-induced reduction in the mitochondrial membrane potential that was dose-dependent and had a progressive character [57]. Moreover, luteolin can influence the pathways via which chemotherapeutics affect cancer cells. In HCT116 human colorectal cancer cells, luteolin induced the Nrf2/ARE/HO-1 activation, which enhances the activity of chemotherapeutic agents, such as oxaliplatin. During the research conducted on the described cells, luteolin caused apoptosis, and oxaliplatin caused the cell cycle arrest, both in a p-53-dependent way. The Nrf2/ARE/HO-1 activation induced by the luteolin-regulated oxaliplatin-promoted p53 signal transduction disturbed the cell cycle arrest. In this way, luteolin strengthened the oxaliplatin’s anticancer activity [56]. A similar observation comes from the research on gastric cancer cells, where mouse forestomach carcinoma (MFC) cells were administered with oxaliplatin and luteolin. This resulted in a synergistic effect due to which cell proliferation was inhibited by the G2/M cell cycle arrest and apoptosis. In the described study, luteolin combined with a low oxaliplatin dose also reduced the mitochondrial membrane potential and led cells to apoptosis (Figure 5) [58]. The described studies prove that luteolin clinical application in the role of a chemotherapeutics enhancer might be significantly effective.

**Figure 6:**
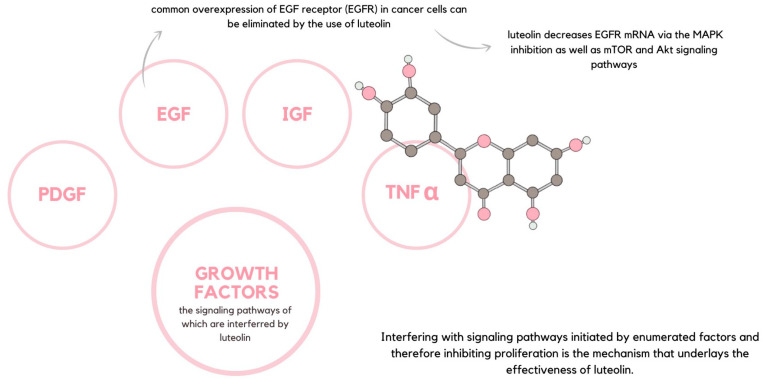
Graphical presentation of the most important growth factors and signaling pathways affected by luteolin, with an example of EGFR overexpression [51,52].

Regarding the suppression of growth factor receptor-mediated cell proliferation signaling, luteolin exhibits dualistic potential. The flavonoid not only interferes with varied components of the signaling pathway but also targets the cell survival pathways, such as MAPK or NF-κB. For this reason, luteolin inhibits cell proliferation and simultaneously promotes apoptosis. Growth factors constitute bioactive molecules, the most common of which are platelet-derived growth factor (PDGF), epidermal growth factor (EGF), and insulin-like growth factor (IGF), as well as TNFα, which can promote the proliferation of cancer cells via the NF-κB pathway. Interfering with signaling pathways initiated by the enumerated factors and inhibiting cell proliferation are the mechanisms that underlay the effectiveness of luteolin (Figure 6) [59]. For instance, the effects of the common overexpression of EGF receptor (EGFR) in cancer cells can be eliminated by the use of luteolin, which decreases the EGFR mRNA via the MAPK inhibition as well as the mTOR and Akt signaling pathways [60].

## 4. Apoptosis Induction

**Figure 7:**
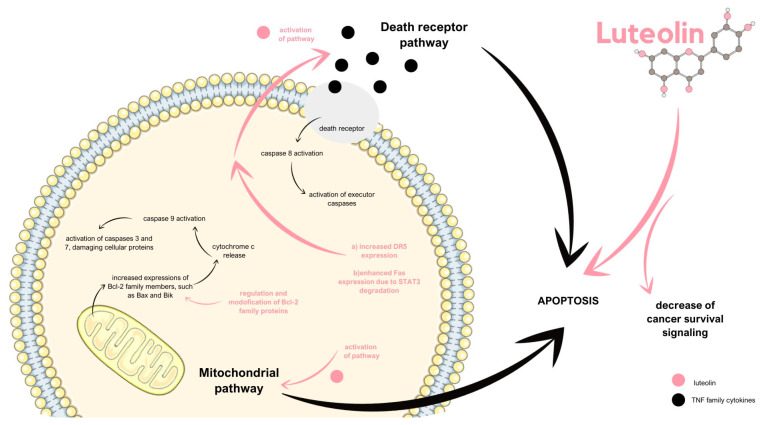
Schematic visualization of possible scenarios of apoptosis as well as the ways in which luteolin is able to interfere with them [53,54,55,56,57,58].

Apoptosis, which means programmed cell death, constitutes a guardian of homeostasis that plays a crucial role in the cell proliferation control system, allowing the elimination of unnecessary or harmful cells, for instance, those with irreversible DNA damage. Defects in apoptotic pathways, either the death receptor or mitochondrial pathway, may result in tumorigenesis [61]. Importantly, even though both pathways lead to the same effect, their origin, as well as the molecules that are involved in them, differ significantly. The mitochondrial pathway is characterized by increased expression of Bcl-2 family members, such as Bax and Bik, which cause cytochrome c release. This protein activated caspase 9, the activator of executor caspases, caspase 3, and caspase 7, which damage cellular proteins. On the other hand, the death receptor pathway is initiated by the binding between the mentioned receptors and the TNF family cytokines, such as TNFα and FasL. The formed connection activates caspase 8, which constitutes the executor caspase activator [62]. Luteolin promotes cancer cell death by enhancing apoptosis and decreasing cancer survival signaling. When it comes to the induction of the apoptotic pathways, luteolin can activate both the mitochondrial and death receptor pathways. On the one hand, the flavonoid induces DNA damage by inhibiting the DNA topoisomerases and activating the p53 protein [47,52]. Additionally, luteolin causes the c-Jun N-terminal kinase (JNK) activation, which leads to the JNK-mediated activation of p53 and expression of Bax, which are responsible for the apoptotic cascade of events. Luteolin also regulates and modifies the expression levels of the Bcl-2 family proteins, which play significant roles in apoptosis induction by the activation of certain caspases. Cancer cells commonly overexpress the prosurvival *Bcl-2* gene, thus preventing apoptosis. Luteolin exhibits the ability to reduce the prosurvival Bcl-xL protein expression as well as to increase the cleavage and activation of caspases, which promotes apoptosis [63]. On the other hand, luteolin enhances the Fas expression due to the pathway in which it promotes the degradation of STAT3, the negative regulator of the *fas* gene transcription. Thus, it provokes the death receptor apoptotic pathway. What is more, 3′,4′,5,7-tetrahydroxyflavone causes an increase in the death receptor 5 (DR5) expression, which constitutes a receptor for TNF-related apoptosis-inducing ligand (TRAIL). This process is possible due to *dr5* gene transcription activation [64]. Not only does luteolin activate the apoptotic pathways, but it also exhibits the ability to suppress signaling that promotes cell survival. For instance, luteolin inhibits the NF-κB survival pathway, thus enhancing the apoptotic effect of TNFα and TRAIL (Figure 7) [65,66]. A recent study on gastric cancer cells revealed that the way luteolin leads the cells to apoptosis is multifactorial. It proved that molecular events such as the destruction of the membrane potential of mitochondria, downregulation of the activity of protein complexes I, III, and V, and thus the impairment of the ATP generation accompany the process of unbalancing the Bcl-2 family members’ expression [67,68].

## 5. Carcinogen Activation

Flavonoids, including luteolin, suppress mutagenic activation of carcinogenesis as they inhibit enzymes belonging to the P450 family, most significantly CYP1A1, CYP1A2, and CYP1B1. The described suppression results in a decrease in the concentration of active mutagens. Thus, metabolism by P450 enhances the antiproliferative potential of flavonoids, which was verified in breast cancer (BC) cells [69].

## 6. Angiogenesis

Angiogenesis is initiated by angiogenic factors, among others, matrix metalloproteases (MMPs) and vascular endothelial growth factor (VEGF). Luteolin constitutes a potential inhibitor of this process, as it can suppress VEGF secretion. The discussed flavonoid promotes the p53-mediated proteasomal degradation of hypoxia-inducible factor-1α (HIF-1α), which triggers the VEGF gene expression. What is more, luteolin also prevents VEGF receptor activation and the initiation of pathways activated by VEGF. Not only does 3′,4′,5,7-tetrahydroxyflavone act directly through VEGF-inducted signal blockade, but it also stabilizes hyaluronic acid, which constitutes a natural barrier for the growth of new vessels by inhibiting the activity of hyaluronidase [70].

Additionally, luteolin was proven to suppress the growth arrest-specific protein 6 (Gas6)-induced the proliferation, migration, and invasion of human microvascular endothelial cells (HMEC-1s) in vitro. The described effect is associated with the inhibition of the Gas6/Axl receptor tyrosine kinase pathway as well as its subsequent phosphatidylinositol 3-kinase/protein kinase B/mammalian target of rapamycin (PI3K/Akt/mTOR) signaling pathways [71].

The invasion and migration to distant localizations of the organism are possible for cancer cells due to the vascular basement membrane degradation, which is caused by type IV collagenases, MMPs [72]. While the MMPs promote invasion, tissue inhibitors of metalloproteinases (TIMPs) prevent it [73]. Luteolin can potentially decrease the levels of MMP-2 and MMP-9 and simultaneously increase the TIMP-1 and TIMP-2 concentrations. Thus, the provoked alterations in the MMP/TIMP balance constitute the mechanism of luteolin’s antimigratory properties [60].

## 7. Metastasis

Luteolin presents potential antimetastatic activity in estrogen receptor (ER)-negative breast cancer, as it constitutes a ligand for the aryl hydrocarbon receptor (AhR), a hope-awakening antimetastatic molecular target. In this way, the flavonoid decreases the expression of MMP-2, MMP-9, and CXC chemokine receptor type 4 (CXCR4), which are prometastatic markers, which was validated during an in vivo examination [74]. What is more, recent studies proved that highly metastatic triple-negative breast cancer (RNBC) pretreatment with luteolin inhibits cell invasion and reverses epithelial–mesenchymal transition (EMT) in a dose-dependent manner. An in vivo model confirmed that the flavonoid can decrease the EMT molecule expression and suppress the lung metastases of breast cancer. Additionally, luteolin decreased the β-catenin mRNA and protein cellular concentrations, suggesting that the EMT reversal might be mediated by the β-catenin downregulation pathway [75]. Apart from those examples, luteolin’s activity generally exhibits the antimetastatic potential. For instance, the fact that this flavonoid decreases the secretion of cytokines such as TNFα, which stimulate metastasis, proves its importance in this field. A similar rule applies to the suppression of the EGFR and NF-κB signaling pathways and regulation of the MMP and hyaluronidase activity [15].

## 8. Breast Cancer

Breast cancer is the leading most commonly diagnosed cancer, with a total of 2.3 million new cases in 2020 [76]. Despite significant advances in its diagnosis and treatment, the disease remains a significant public health challenge. Researchers are constantly exploring new therapeutic options to improve breast cancer patients’ treatment outcomes and quality of life. Phytochemicals, such as biochanin-A, diindolylmethane, emodin, curcumin, lycopene, rosmarinic acid, epigallocatechin gallate, resveratrol, genistein, sulforaphane, shikonin, rutin, and silibinin, have been extensively studied for their potential in breast cancer treatment. Among these, polyphenols, including luteolin, have gained significant attention due to their ability to induce apoptosis and autophagy, anti-inflammatory effects, and maintenance of redox balance. As a result, they have become a prominent subject of research. Luteolin significantly impacts cellular functioning. In breast cancer cells of different subtypes, it was found to [77]:

- Block the proliferation of IGF-1-stimulated luminal A subtype ERα-positive MCF-7 cells;
- Suppress the growth of triple-negative/basal-like ERα-negative MDA-MB-231 cells;
- Decrease the viability of breast cancer cells MCF7/6 and MDA-MB231-1833;
- Reduce the tumor burden in nude mice inoculated with MDA-MB-231 cells;
- Inhibit the migration and invasion of highly metastatic triple-negative breast cancer (TNBC) cell lines MDA-MB-231 and BT-549;
- Suppress the formation of lung metastases in breast cancer xenograft tumors originating from MDA-MD-231 cells;
- Inhibit the migration of ERα-positive MCF-7 cells;
- Inhibit the migration and viability of human MDA-MB-435 and MDA-MB-231 cells;
- Reverse the epithelial–mesenchymal transition (EMT) of MDA-MB-231 and BT5-49;

The most effective mechanism against breast cancer may be the modulation of apoptosis and angiogenesis. Luteolin induces apoptosis in MDA-MB-231 breast cancer cells by inactivating the caspase cascade and PARP. This action promotes these cells’ extrinsic and intrinsic apoptotic pathways [78]. Luteolin also blocks cell proliferation, hinders cell cycle progression, and triggers apoptosis within 24 and 48 h of analysis [79]. What is more, a recent study revealed that luteolin significantly downregulates the expression of four genes (AP2B1, APP, GPNMB, and DLST), which are associated with drug resistance, cell proliferation, macrophages, apoptosis, and HDAC inhibition and are highly expressed in breast cancer cells [80]. Significant studies on luteolin’s application in breast cancer were conducted on triple-negative breast cancer (TNBC), which accounts for around 15–20% of all breast cancer cases diagnosed, is more commonly found in younger, African-American women, and is characterized by the lack of estrogen receptor (ER), progesterone receptor (PR), and human epidermal growth factor receptor 2 (HER2) expression. As a result, this subtype is resistant to currently available receptor-targeted therapies [79]. Luteolin was found to significantly inhibit the cell proliferation and metastasis of TNBC, which was linked to the inactivation of the AKT/mTOR signaling pathway and reversed EMT [72]. Cisplatin was found efficient against TNBC associated with BRCA1, and luteolin can protect against nephrotoxicity caused by cisplatin as it decreases the p53-mediated renal tubular apoptosis, making it a promising agent for combination therapy with cisplatin [81]. AR-positive TNBC cells exhibit a greater susceptibility to luteolin than AR-negative TNBC cells [72]. An analysis of the mechanisms behind luteolin treatment has shown that it significantly increases the production of intracellular ROS in TNBC cells but not in normal breast epithelial cells, and that the growth-inhibitory effect of luteolin on TNBC cells is attenuated by the use of a radical oxygen scavenger called N-acetyl cysteine, indicating that ROS generation may play a role in the observed growth inhibition. Another proposed mechanism is activating the p53 signaling pathway and increasing the heat shock protein 60 levels in response to luteolin treatment, which may partly contribute to the observed effect on the TNBC cell growth [79].

### 8.1. MicroRNAs

MicroRNAs (miRNAs) are a class of small non-coding RNAs that play a role in the regulation of protein synthesis and stability of mRNAs as well as in the initiation and progression of breast cancer [82]. Due to their involvement in tumor cell growth and metastasis, some miRNAs have been identified as oncogenic or antioncogenic [77,83]. MiR-203 exerted contradictory roles in breast cancer [83]. A recent study on luteolin’s properties to fight breast cancer showed that it increased the expression of miR-203, and its antitumor effects were partially eliminated when the miR-203 expression was silenced. It also proved that luteolin’s inhibitory effect on Ras/Raf/MEK/ERK signaling (critical signaling in driving the initiation of cancers) was reduced or even reversed when miR-203 was suppressed. Overall, luteolin was found to reduce breast cancer cell growth and EMT significantly, and its antitumor effects may be due to elevated expression of miR-203 and inhibited Ras/Raf/MEK/ERK signaling [83]. A previous study demonstrated that luteolin exposure resulted in higher levels of certain rarely observed miRNAs, such as miR-34a, miR-181a, miR-139-5p, miR-224, and miR-246, in breast cancer cells, and miR-155 levels were significantly lower. Furthermore, when synthetic copies of miR-34a or miR-224 were introduced into the cells, a direct decrease in the Notch-1 levels was observed. This suggests that luteolin may inhibit Notch signaling by regulating miRNAs. The excessive activation of Notch signaling has been widely acknowledged as a critical factor contributing to cancer cells’ ability to survive, grow, spread, move, and develop new blood vessels [82].

### 8.2. IGF-1 Pathways

The insulin-like growth factor 1 (IGF-1) pathways, including MAPK and PI3K, play a crucial role in the formation of tumors [84]. The IGF-1 pathways impact breast cancer growth but are also vital for normal mammary gland biology, and the breast cancer risk and progression arise when the dysregulation of the IGF-1 receptor’s expression occurs, which was proved by experimental findings showing that IGF-1 significantly induces the growth of MCF-7 breast cancer cells [85]. While luteolin has been found to exhibit estrogen agonist activity, it also exerts antiestrogenic effects by the modulation of multiple genes involved in the estrogen signaling pathways such as GTF2H2, NCOR1, TAF9, NRAS, NRIP1, POLR2A, DDX5, and NCOA3 in breast cancer cells, possibly through an epigenetic mechanism [77]. The IGF-1 signaling pathways impact breast cells’ response to estrogen since IGF-1 and insulin at supraphysiological concentrations can enhance the estrogen receptor (ER) activation by estrogen. IGF-1 can also cause direct phosphorylation of ER by PI3K/AKT, mTOR, and MAPK signaling. Moreover, this relationship turns out to be bidirectional since the ER stimulation upregulates the expression of the IGF-1 receptor (IGF-1r), and estrogen stimulates the binding of ER to the IGF-1r, which promotes downstream signaling [85]. The luteolin’s inhibitory effect on the IGF-1-induced breast cancer cell growth turned out to be dependent on ER α since the knockdown of ER α decreased the inhibitory effects of this substance on the IGF-1-induced cell growth [86]. Luteolin has also been found to decrease the ER α expression and effectively inhibit the growth of the IGF-1-stimulated MCF-7 cells [79]. Luteolin effectively blocks the rapid proliferation of luminal A subtype ERα-positive MCF-7 breast cancer cells stimulated by IGF-1 in a dose- and time-dependent manner [86]. These findings offer a promising new therapeutic approach to address the challenges in breast cancer treatment.

### 8.3. Kinases 

Polo-like kinase 1 (PLK-1), a kinase that plays a crucial role in cell division, is overexpressed in many types of human cancers, which is associated with a poor prognosis. Luteolin has been found to inhibit the expression of the PLK1 gene in both MCF-7 breast cancer cells and MDA-MB-231 ER-negative breast cancer cells, which is likely due to a decrease in the acetylation of histone H4 associated with the promoter region [77]. Protein Kinase B (PKB), also known as Akt, is a serine/threonine-specific protein kinase that is well established as a critical signaling node in the regulation of cellular proliferation, survival, apoptosis, and metabolism, which are also the processes important for tumorigenesis [87]. The FOXO transcription factor subfamily, a member of the larger forkhead family of transcription factors, is critically involved in determining cell fate and has been implicated in a significant number of cancers [88]. The Akt/FOXO3a signaling pathway is highly involved in regulating various cellular processes implicated in cancer development and progression [89]. The FOXO pathway is closely monitored by the PI3K/AKT signaling pathway, which is crucial for cell growth and proliferation [88]. The overexpression of FOXO3a has been found to be associated with Akt phosphorylation, which can impact the prognosis of breast cancer [79]. Luteolin can cause breast cancer cells’ cycle arrest and apoptosis by blocking the activation of the PI3K/Akt pathway and increasing the activation of FOXO3a [77]. This action yields significant outcomes, such as preventing the penetration of breast cancer spheroids through the lymph endothelial barrier [79]. Also, at a concentration of 20 µM, luteolin downregulates the GPNMB gene expression, which triggers the self-renewal of spheroids to increase cell survival, and its activity is sufficient for tumor spheroid formation [80]. Luteolin at various doses from 1 to 2 μM was found to suppress the expression of nuclear factor erythroid 2-related factor 2, heme oxygenase 1, and cripto-1, which have been linked to the functioning of cancer stem cells. Cancer stem cells are a subpopulation of cancer cells that bear the capacity for self-renewal, cancer recurrence, and resistance to chemotherapeutics [90]. Therefore, luteolin is suggested to reduce breast cancer stemness.

## 9. Chemosensitization

Luteolin is effective in multidrug-resistant (MDR) cancer by inducing apoptosis in MDR cancer cells by generating reactive oxygen species, inhibiting the NF-κB pathway, and depleting the antiapoptotic proteins [79]. Moreover, it can enhance the efficacy of breast cancer treatment drugs by acting as a chemosensitizer for anticancer agents. This effect has been reported in several studies, some of which are described below.

### 9.1. Doxorubicin

Most solid tumors that develop in a clinical setting tend to thrive in an environment that lacks sufficient oxygen, known as hypoxia. Unfortunately, cancer cells have the ability to adapt to low-oxygen conditions, which makes them less responsive to commonly used anticancer medications. Hypoxia can increase the resistance of cancer cells to chemotherapy by 2–5 times [91,92]. Luteolin at 50 or 100 μM under hypoxic conditions has been found to enhance the cytotoxicity of doxorubicin, reverse hypoxia-conferred tumor resistance to doxorubicin, and enhance apoptosis induced by doxorubicin. The combined treatment of luteolin and Dox was effective even without the immune system’s involvement in killing cancer cells [92]. The success of this combination therapy is largely attributed to the inhibition of glycolysis in breast cancer cells, which enhances the anticancer effects of Dox while minimizing its toxicity. On the other hand, luteolin at low concentrations (as low as 10 μM) was found to attenuate doxorubicin-induced cytotoxicity in breast cancer cells, probably through a combination of antioxidant activity and an increase in the levels of Bcl-2 protein, and breast cancer cells’ viability decreased when luteolin concentrations were increased (>30 μM) [91]. The appropriate dosage of luteolin is a crucial factor in determining its impact on breast cancer.

### 9.2. Celecoxib

Numerous studies have been conducted to investigate the antitumor effects of celecoxib and luteolin in the treatment of breast cancer when used individually. This combination treatment significantly reduced cancer cell viability, with greater efficiency in killing tumor cells observed after 72 h of treatment. The combination of celecoxib (at concentrations of 50 or 100 μM) and luteolin (at concentrations of 25, 50, 75, and 100 μM) provided superior inhibition of breast cancer cell growth and an increase in breast cancer cell apoptosis greater than after either celecoxib or luteolin treatment alone [93]. This treatment led to a noteworthy decrease in Akt phosphorylation, and breast cancer has been frequently reported to exhibit increased Akt phosphorylation [79]. These results suggest that a combination of celecoxib and luteolin may be a new possible treatment option for breast cancer.

### 9.3. Cyclophosphamide

In rats, the combined treatment of luteolin (30 mg/kg) and cyclophosphamide (10 mg/kg) was found to significantly reduce tumor volume without any toxicity from cyclophosphamide, likely due to the potent antioxidant properties of luteolin. Moreover, the combination of luteolin and cyclophosphamide demonstrated robust chemopreventive activity against developing DMBA-induced mammary tumors.

**Figure 8:**
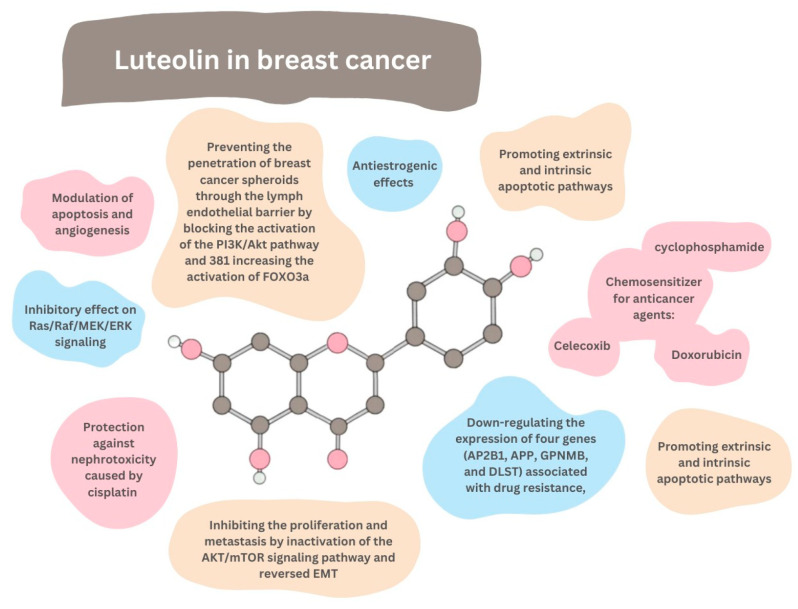
Schematic visualization of the multitude of roles that luteolin plays in BC [68,69,70,71,72,73,74,75,76,77,78,79,80,81,82,83,84,85,86,87].

Luteolin at 30 μM was found to cause a dose-dependent cell cycle arrest in the G2/M and S phases, increase the apoptotic rates, and suppress the proliferation of breast cancer cells in a dose-dependent manner [94]. A study on breast cancer cells found that luteolin inhibited the phosphorylation of nuclear factor-κB inhibitor α and its target gene c-Myc, which led to the suppression of the human telomerase reverse transcriptase expression, the catalytic subunit of telomerase [94]. When luteolin was administered orally, no signs of toxicity were observed in relation to the body’s overall weight [8]. On the other hand, In an epidemiological study investigating the correlation between the dietary intake of luteolin and the anticancer effects, no significant association was observed [95]. This lack of correlation could be attributed to the low daily luteolin intake, which was less than 2.0 mg/day. In contrast, therapeutic doses or concentrations of luteolin used to achieve significant anticancer effects can range from 10 to 30 mg/mL [77]. Further investigation could provide valuable insights into how tumor cells respond to chemotherapy drugs in various environmental conditions and could lead to the development of a promising therapeutic approach for effectively eliminating cancer cells (Figure 8).

## 10. Colon Cancer

Colon cancer constitutes one of the most common neoplasms in the world. It is the third most common cancer in men and the second most common cancer in women. Colon cancer is also one of the most common causes of cancer-related deaths [96]. When discovering the connection between luteolin and colon cancer, it is crucial to notice that luteolin affects the interleukin 6 signaling pathway [97]. Interleukin 6 is an activator of STAT3, which regulates the course of several cancers, including colorectal cancer [98]. Importantly, an inflammatory environment promoted by cancer may lead to the activation of the interleukin 6 signaling pathway, and this can cause metastases [99]. In one of the studies aiming to determine the way in which luteolin affects tumor cells, human colon cancer cell lines SW480 and SW620 were incubated with THP-1 cells and LPS [97]. The THP-1 cells affected by LPS were stimulated into transformation to macrophages M1, which produce tumor necrosis factor and interleukin 6 [100]. The results showed that the levels of Interleukin 6 were much higher in the group with macrophages stimulated by LPS than in the control group. On the other hand, in groups where macrophages were incubated with LPS, and luteolin was added, the level of IL-6 was significantly lower [97]. The study also revealed that M1 macrophages promote the phosphorylation of STAT3 and cell proliferation, migration, and invasion. All of these processes are inhibited by luteolin. In addition, a series of experiments conducted with IL-6 and IL-6 antibodies revealed that IL-6 induces cell proliferation, migration, and invasion; however, IL-6 antibodies do not affect cancer cells. On the other hand, when incubating cancer cells with IL-6, IL-6 antibodies, and M1 macrophages, anti-IL-6 antibodies partially inhibit the effect that macrophages have on the cancer cells. In the last experiment, the cancer cells were incubated with M1 macrophages which were treated with luteolin. Then, interleukin 6 was added to one group, and anti-IL-6 antibodies were added to the other. Luteolin inhibits M1 polarization which is reversed by the IL-6, whereas the anti-IL-6 antibodies enhance the positive effect of luteolin. The study therefore revealed that luteolin can affect the IL-6 signaling pathway and in the end reduce the cancer cell proliferation and metastases [97]. So, it may be possible to use luteolin in future to slow the progress of the disease. Another study focused on how luteolin can affect process of autophagy and apoptosis in colon cancer cells [53]. The P53 gene’s main role is to repair DNA, but it can also activate apoptosis and autophagy when cell damage is too severe to be repaired. Because of these qualities, patients with mutant P53 have poorer prognosis than patients with standard P53 [101]. In another study, three types of cells were cultured: HCT116, which are colon cancer cells with wild-type P53; HT-29, which are colon cancer cells with mutant P53; and normal colon fibroblasts, CCD-18Co. The results showed that the viability of HCT116 cells treated with luteolin was lower than that in the control group; however, the luteolin treatment did not have any effect on the HT-29 and CCD-18Co cells. To determine if the apoptosis is responsible for the viability loss, the PARP protein was measured. PARP is a marker of apoptosis, and significantly higher levels of it were measured in the HCT116 cells treated with luteolin [53]. This outcome strongly suggests that luteolin can activate the apoptosis and reduce the proliferation of cancer cells. However, luteolin can affect only cells without mutant P53. To test how luteolin can affect autophagy, the levels of autophagy markers were controlled. Such markers are LC3-II and Beclin 1, and both of them were elevated in the HTC116 cells [53]. This suggests that luteolin increases the process of autophagy. To test this theory, CQ, an autophagy inhibitor was added to the cells. It stopped autophagy, but the overall level of cytotoxicity and apoptosis was the same as that within the cells treated with luteolin but not with the CQ. This suggests that luteolin may induce autophagocytosis but not enough for it to have any meaningful effect on cells. The overall results suggest that luteolin can affect the progression of colon cancer and help with the treatment of this disease; however, more research needs to be conducted in order to understand the full potential of luteolin [53]. Similar results were achieved in a different study which focused on the way luteolin affects SW620 cells [102]. The first experiments indicated that luteolin induces apoptosis which was proved by higher expression of Bax, which is a proapoptotic protein, and lower expression of BCL-2, which is an antiapoptotic protein. The following experiment confirmed that luteolin induces autophagy by detecting higher levels of Atg7, Beclin-1, and LC3B-I and LC3B-II in cells treated with luteolin. However, a hypothesis was made that the autophagy induced by luteolin has a cytoprotective role and, overall, lowers the apoptosis of cancer cells. To test this theory, cells were incubated with autophagy inhibitor 3-MA. The results showed that the administration of 3-MA lowered the viability of cells, therefore confirming the hypothesis of the cytoprotective role of autophagy. In addition, the experiments showed that luteolin causes oxidative stress in cells by exposing higher levels of antioxidant enzymes such as Ho-1 and SOD2 [102]. Another important thing is that luteolin inhibits the epithelial–mesenchymal transition of colon cancer cells. It was determined by detecting lower expression of the Wnt3 and β-catenin proteins in cells treated with luteolin. Both of these proteins are involved in the EMT [102,103,104]. Another interesting discovery was that luteolin increases the expression of FOXO3a and phospho-FOXO3a in SW620 cells [102]. FOXO3a is connected with the inhibition of cell proliferation and apoptosis, so it has anticancerogenic activity. However, the phosphorylation of FOXO3a makes it inactive, promoting the initiation and proliferation of cancer [105]. Even though luteolin increases the levels of active and inactive FOXO3a, the level of active FOXO3a is increased by a higher amount, and, therefore, its anticarcinogenic activity is exhibited more. Luteolin has been observed to activate ERK1 and ERK2 in SW620 cells. Together with P38, which is also activated by this flavonoid, they increase apoptosis, which was confirmed by an increased concentration of caspase-3. Additionally, luteolin can reverse the process of the epithelial–mesenchymal transition [102]. This effect is achieved through two different pathways: WNT/β-catenin and FOXO3a. Both of these pathways have documented effects on the EMT [103,104]. Luteolin suppresses the WNT/β-catenin pathway and increases the levels of FOXO3a, reversing the epithelial–mesenchymal transition process [102]. Such results can only reinforce the belief that luteolin can be used as an effective drug in the fight against colon cancer; however, more tests need to be performed to assess the real-life application of luteolin, especially in clinical trials. A different study focused on the effect of luteolin on hypoxia-inducible factors and revealed that luteolin significantly decreased the levels of hypoxia-inducible factors compared to cells that did not have contact with luteolin [106]. Hypoxia-inducible factors are one of the main cell products that can alter cell metabolism to adapt to an environment with a lower oxygen level. Such factors can be found in colon cancer [107]. They serve as transcription factors that, under normal conditions, are hydroxylated, which causes their degradation [106]. However, when partial oxygen pressure drops under 2%, the enzymes that are responsible for the hydroxylation of these factors become disabled, and this allows for the accumulation of hypoxia-inducible factors that transactivate genes and alter the metabolism of cells [106]. Additionally, these factors can induce the epithelial–mesenchymal transition, which is an important factor in the metastatic potential of the tumor [108,109]. In the light of how these factors can contribute to the survival of cancer cells in unfavorable conditions as well as how they can induce immune evasion and metastasis, it is important to find substances with such capabilities. Luteolin is a promising drug in future therapy of colon cancer, especially because it has a broad range of activities that lower cancer cell survival. However, to fully assess the usefulness of luteolin in therapy, more research needs to be conducted focusing on living organism and, if these experiments bring promising results, clinical trials on humans need to be performed. 

## 11. Lung Cancer

**Figure 9:**
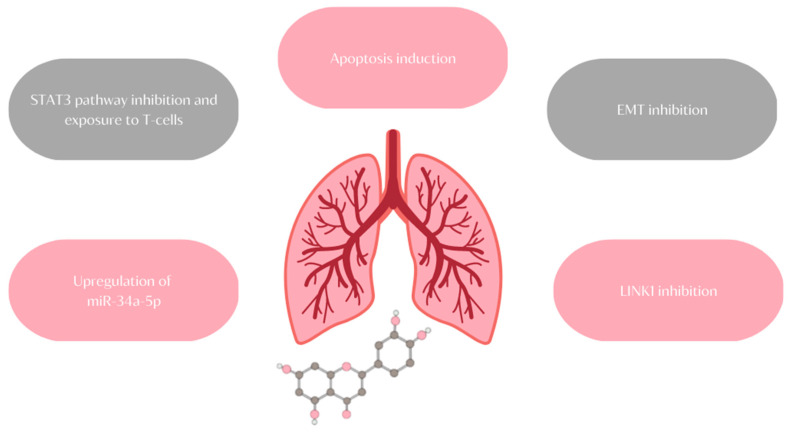
Schematic visualization of the molecular pathways that luteolin affects, therefore contributing to lung cancer growth inhibition [31,117,118,119,120].

Lung cancer is the second most diagnosed cancer in both men and women and is the leading cause of cancer death worldwide [110]. There are two main types of lung cancer: small cell lung cancer (SCLC) and more common non-small cell lung cancer (NSCLC) [111]. Various treatment options are available, but the outcome of therapy depends on the type, stage, number of metastases, and time of diagnosis [112]. Early diagnosis gives more therapeutic options and leads to a higher survival rate [113]. However, lung cancer is paucisymptomatic or even asymptomatic at its initial stages, so the diagnosis is usually made when the cancer is at advanced stages [113,114]. As lung cancer is still a major problem worldwide, it is still crucial to search for new diagnostic and treatment options [115]. Luteolin exhibits anticancer properties, so it may be a new effective agent against lung cancer (Figure 9) [116].

Numerous gene mutations are relevant to carcinogenesis, so exploring and studying these mutations play key roles in treatment research. There are over 1000 mutations connected to lung cancer, but EGFR, TP53, ERBB2, CDKN2A, and KRAS are the most common well-known genes for NSCLC [121]. KRAS mutation increases the expression of programmed death ligand 1 (PD-L1) in cancer cells, enabling the tumor to evade the immune system [122]. An in vitro study showed that luteolin inhibits the proliferation of KRAS-mutant cells in a dose-dependent manner. The PD-L1 expression is a result of the STAT3 activity, which, after phosphorylation, binds to the PD-L1 promoter. Luteolin inhibits the STAT3 pathway and PD-L1 expression, which exposes the tumor cells to T cells [122]. In various cancers, including lung cancer, there is a great deal of different microRNA (miRNA) dysfunctions [123]. The role of miRNAs in cells is to regulate gene expression, and in cancer cells, they may have pro- or antioncogenic effects depending on the type of miRNA [124]. For example, miRNA-195 inhibits lung cancer progression, but it is promoted by miRNA-196b-59 [124]. Particularly interesting is the role of the miR-34 family in different kinds of lung cancer, as its expression in tumor tissue enables the prediction of a patient’s survival [125]. High level of miR-34a suppresses tumor growth; therefore, it is downregulated in cancer cells [126]. Studies by Jiang Z-Q et al. demonstrated that luteolin upregulates miR-34a-5p, and, thereby, it may inhibit NSCLC cell proliferation [127]. 

The luteolin’s anticancer properties are also exhibited by inducing apoptosis in cancer cells [128]. There are two main apoptotic pathways, extrinsic and intrinsic, called the mitochondrial pathways [129]. Luteolin promotes the activation of Caspase-9 and Caspase-3, which consists of the intrinsic pathway activation [128]. Furthermore, in vitro studies show changes in the level of the Bcl-2 and BAX proteins that are also involved in the mitochondrial pathway of apoptosis [130]. Bcl-2 is an antiapoptotic protein, while BAX, on the contrary, is a proapoptotic factor [129]. Luteolin decreases the Bcl-2 expression and increases that of BAX, so that BAX/Bcl-2 ratio increases and induces apoptosis [130]. What is more, the luteolin-induced accumulation of reactive oxygen species(ROS) can activate a TNF-induced model of the extrinsic pathway [131]. An especially significant step in the cancer progression is the epithelial–mesenchymal transition (EMT), when epithelial cells transform into mesenchymal stem cells and, as a result, gain invasive properties [118,128]. As a matter of fact, it is a process wherein benign tumor becomes invasive [118,128]. A cadherin switch occurs during this process, so E-cadherin is replaced by N-cadherin [118]. There is a correlation between the E-cadherin level in cancer cells and overall survival—the higher the E-cadherin expression, the better the prognosis since the E-cadherin level is decreased in invasive tumors [118]. The EMT and E-cadherin downregulation is activated by TGF-β1 and the PI3K/AKT-NF-κB-Snail signaling pathway [120]. Luteolin can inhibit the EMT by suppressing the previously mentioned factors [128]. Another promalignant agent is LIM kinase 1 (LIMK1) [119]. The LIMK1 phosphorylation results in the stimulation of angiogenesis, cancer cell proliferation, and metastasis progression. It may be used as a prognostic marker since the upregulation of LIMK1 mRNA may indicate lymph node metastases [119]. Another study showed that LINK1 is a target of luteolin, and by inhibiting the LINK1 activity, luteolin may inhibit the tumor growth [55]. Not only is luteolin an interesting candidate for new anticancer drugs, but it may also be an analgesic for reducing lung cancer-induced bone pain [31]. Bone metastases are highly common for lung cancer, and in 80% of patients, the first symptom of osseous invasion is severe pain [132]. The exact mechanism of bone cancer pain remains unknown, but some studies demonstrated that neuroinflammation, glial activation in the spinal dorsal horn (SDH), and abnormal function of the NOD-like receptor protein 3 (NLRP3) inflammasomes participate in bone pain development [31]. A study by Zhou Y-S et al. revealed that luteolin targets SDH and inhibits the activation of glial cells and NLRP3 inflammasomes [31]. It also demonstrated that luteolin is able to cross the blood–brain barrier; hence, it may act and produce an analgesic effect in the central nervous system, although it has not been proven [31]. Luteolin influences many factors and pathways concerning lung cancer development. It may expose tumor cells to immune system activity, induce apoptosis through different pathways in cancer cells, and inhibit cell proliferation and malignant transformation. Besides its anticancer properties, worth mentioning is also the ability to relieve bone cancer pain [31]. Nevertheless, there are only in vitro and in vivo studies, and the effect of luteolin on lung cancer in humans is still unknown.

## 12. Inflammatory Skin Diseases

Luteolin is known for its antioxidative and anti-inflammatory properties, which impact keratinocytes, fibroblasts, and several immune cells such as mastocytes, neutrophils, or dendritic cells [32]. By repressing the proinflammatory response, which is performed through the suppression of mediators (such as IL-1β, IL-6, or TNF-α) and regulation of various signaling pathways (e.g., the JAK-STAT, TLR pathways, or NF-κB), luteolin may present quite a positive impact in mitigating many inflammatory processes of the skin [32]. Luteolin can influence the Jak/Stat signaling pathway. The increased JAK signaling leads to diseases such as leukemias or inflammatory illnesses [133]. The flavonoid combined with paclitaxel can substantially decrease the amount of phosphorylated STAT3 in MB-231 cells, resulting in the tumor’s shrinking [134].

By alternating the Nrf2/MAPK signaling, luteolin can protect the cardiac muscle from reperfusion damage. Additionally, reducing the expression of JNK and p38MAPK interrupts the proliferation of cartilage cells in osteoarthritis, which leads to reduced inflammatory responses and postponed cartilage deterioration [135].

By influencing these pathways, luteolin can lower proinflammatory cytokine production, inflammatory cell migration, and inflammatory gene expression [67].

Luteolin has very versatile activity and influences very different pathways, which demands more studies to understand its effect on organisms fully. However, most known mechanisms strongly suggest that luteolin has strong anti-inflammatory activity. Additionally, luteolin affects various immune cells and their qualities; therefore, it shows that luteolin is a substance that alters many processes, not only those connected with inflammation [67].

All this evidence suggests that luteolin might be an anti-inflammatory drug, but more extensive research is required to understand the many complicated ways it affects human biochemistry.

## 13. Psoriasis

**Figure 10:**
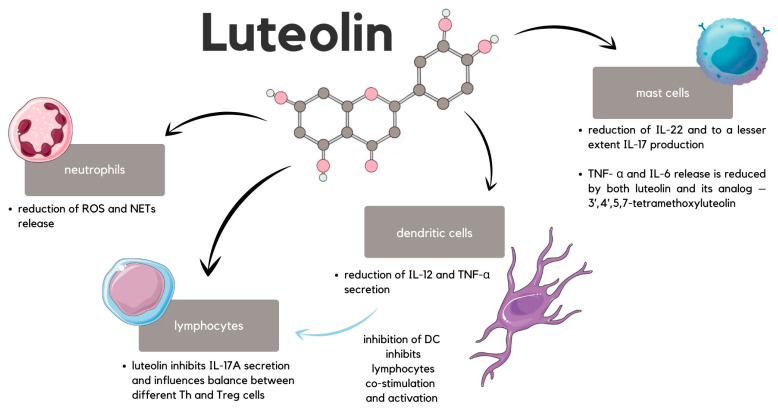
Schematic visualization of the ways in which luteolin influences neutrophils, mast cells, dendritic cells, and lymphocytes [139,140,141].

The first condition that luteolin can modulate is psoriasis. Psoriasis is a chronic inflammatory disease that most commonly manifests itself through the formation of indurated plaques covered by silvery scales and sharply demarcated erythematous—it is a typical clinical depiction of psoriasis vulgaris, constituting approximately 90% of all cases. Globally, psoriasis applies to 2–3% of the population, thus influencing over 125 million people [32]. Based on the known and established general characteristics of inflammatory skin disease, luteolin can be beneficially implemented in these patients. The anti-inflammatory and antioxidant properties of this flavonoid stem from its either direct interaction with signaling molecules or by regulating signaling pathways. Moreover, positive effects are only enhanced by fewer side effects compared to synthetic active substances. The signaling of different TLRs on immune cells consequently plays a significant role in immune reaction in psoriasis. However, before this signaling pathway is attained, unspecific factors (e.g., chemical irritants) trigger the release of nucleic acids—DNA and RNA—from keratinocytes. Nucleic acids form complexes with antimicrobial peptides (AMPs) present in the skin, activating innate immunity cells—neutrophils, mastocytes, or dendritic cells. This activation is attained via Toll-like receptors (TLRs) and is followed by the release of ROS and neutrophil extracellular traps (NETs) from neutrophils, which only aggravates the immune response [136]. Luteolin is crucial in ceasing this immunological sequence since it inhibits the signaling of different TLRs on various cells. This inhibition is achieved by reducing the expression of TLRs and TLR target genes [137], impeding the formation of TLR signaling complexes, or inhibiting signaling molecules [138]. Another aspect of the activity of luteolin is the reduction in ROS and NETs released from the neutrophils [139]. Other immune cells that remain under the influence of luteolin are dendritic cells (DCs) and mast cells. It has been shown in mice that luteolin treatment decrease the number of CD11+ DCs and reduce the secretion of IL-12 and TNF-α by these cells [140]. The significance of this derives from the fact that DCs play an important role in co-stimulation and, thus, in T cell activation. Regarding the mast cells, luteolin suppresses the production of mainly IL-22 and, to a lesser extent, IL-17 [32]. Other proinflammatory cytokines contributing to inflammatory aggravation in psoriasis released by mast cells are TNF- α and IL-6. Luteolin and its analog—3′,4′,5,7-tetramethoxyluteolin—participate in the inhibition of mast cells [141]. Activated DCs and mast cells release cytokines that stimulate the activation and differentiation of T cells into IFN-γ-producing Th1, Th22, and Th17, which secretes proinflammatory IL-17A [32]. Luteolin, however, has an effect on T cell proliferation and secretion of IFN-γ which is particularly important in psoriasis [142]. Moreover, luteolin inhibits the IL-17A secretion and, lastly, it influences the balance between different Th and Treg cells (Figure 10) [140]. In a murine psoriasis model, luteolin decreased the Th1 and Th17 cells while increasing the Th2 and Treg cells [143]. Luteolin is also known to impact macrophages, as LUT-7G reduces the production of proinflammatory PGE2 by these cells after activation. However, its anti-inflammatory properties are also attained by increased production of cortisol [144].

Having established the role of immune cells in the pathogenesis of psoriasis and the positive, mitigating impact of luteolin, other pathways aggravating this disease should be studied. Responsive to the already discussed cytokines, keratinocytes begin to proliferate and trigger proinflammatory pathways [32]. Their proliferation is responsible for the phenotypic outcome of psoriasis and is associated with the IL-22 and IL-6/STAT3 pathways [144]. Therefore, the inhibition of these pathways shall be potentially a good therapeutic target, bringing positive outcomes, and, indeed, it was a research subject of Palombo et al. [144]. It was stated that LUT-7G blocked the STAT3 pathway both in vitro and in vivo in a murine psoriasis model [145]. Moreover, when used concurrently, LUT-7G decreased the proliferation marker expression [144]. Apart from hyperproliferation, keratinocytes in psoriatic skin are also characterized by a lack of differentiation [32]. In vitro studies have shown the ability of luteolin to reduce the number of proinflammatory cytokines (e.g., IL-6, IL-8) by inhibiting the NF-κB signaling [143]. Similar results with additional inhibition of VEGF secretion were achieved by suppressing the mTOR pathways, which was accomplished with a luteolin analog—3′,4′,5,7-tetramethoxyluteolin [146]. A separate study presented the ability of luteolin-7-glucoside (LUT-7G) to increase the number of differentiation markers, thus reducing the block of keratinocyte differentiation in vitro [144]. Interestingly, micromolar concentrations of luteolin-7-glucoside could modify the cell cycle in vitro by promoting differentiation rather than proliferation, which could not be achieved when a different flavonoid, very similar to luteolin, apigenin, was used. Furthermore, a murine in vivo psoriasis model confirmed those results by reducing cell proliferation and scale thickness and increasing the keratinocytes’ differentiation markers [144]. Similar results were reported when luteolin-7-O-β-D-glucuronide was applied [147]. More recent research confirmed these results and proved that luteolin may suppress the production of NO, iNOS, and COX-2, while LUT-7G inhibits the cellular energy production of keratinocytes [148]. Despite promising in vitro and in vivo results, there is still little knowledge about the application of luteolin and its analogs in psoriatic patients—only four patients have been treated with a lotion applied twice a day that contained a luteolin analog (3′,4′,5,7-tetramethoxyluteolin). Nevertheless, they presented beneficial effects on their psoriatic symptoms after using a lotion for a month. After studying another promising contribution of luteolin, it was suggested that LUT-7G in vivo could reverse the inflammatory and cell proliferative phenotype produced by imiquimod (IMQ), which implies that it could be used to treat psoriasis [144]. However, more research is still needed to confirm all these promising results, which may improve the quality of life for patients with this skin disease, especially with fewer side effects compared to standard therapy. 

## 14. Atopic and Contact Dermatitis

Contact dermatitis can be distinguished among inflammatory skin diseases as another example of a disease on which luteolin could have a positive impact. It is either an acute or chronic eczematous skin reaction developing after, usually repeated, exposure to harmful irritants, which most commonly are xenobiotic chemicals [32]. The European Society for Contact Dermatitis differentiates four types of this disease—allergic contact dermatitis (ACD), irritative contact dermatitis (ICD), photo contact dermatitis, and contact urticaria (which remains a type I IgE-mediated allergic reaction). However, the first type, ACD, which affects approximately 20% of the population [32], is more extensively studied in this work. It is worth mentioning that ACD remains a classical type IV T cell-mediated allergy, and, consequently, it presents with the activation of the innate immune system and adaptive immune response, which is opposite to ICD, in which the adaptive immune response is not activated [149]. Apart from the extensive focus on the pathogenesis and possible treatment of ACD, valid therapeutic options are yet to be discovered as, unfortunately, the gold standard is avoiding possibly causative allergens and applying corticosteroids. Nevertheless, the application of luteolin derived from *Reseda luteola* (L.) was studied to determine whether it could reduce the inflammation in these diseases. The subjects were 25 healthy volunteers who applied cream containing luteolin to their skin previously irritated with repeated washing with sodium lauryl sulfate, which significantly diminished the redness of the skin, and, moreover, the skin was more hydrated with a much smaller transepidermal water loss [150]. Interestingly, after using the same extract, UVB-induced skin inflammation in vivo was reduced [151]. These conclusions suggest that luteolin, with its antioxidative activity, may prevent skin irritation resulting from frequent washing and use of water-soluble irritants as well as from UV exposure [32]. Another mechanism promoting ACD development, namely the sensitization phase of ACD, is the production of ROS by contact sensitizers, and in a murine contact hypersensitivity (CHS) model of ACD, the suppression of ROS formation by antioxidants such as luteolin was able to abolish both the sensitization and elicitation phases [32,152]. Different studies analyzed *Bryophyllum pinnatum* (Lam.)—a plant that contains flavonoids rutin, quercetin, and luteolin 7-O-β-d-glucoside alongside luteolin. Although the direct effect of luteolin could not be analyzed, the ethanol leaf extract that was obtained successfully reduced irritant–induced ear edema in mice [153]. A similar effect, reduced ear swelling, was obtained after the application of hydrogel formulation with CYR (cymaroside, known as luteoloside) extracted from *Bidens tripartite* (L.) that contained 7-O-glucoside of luteolin. This positive outcome supports the thesis that flavonoids such as luteolin can be used to suppress innate immune responses from stem inflammatory conditions such as irritative dermatitis. Interestingly, the positive effect of CYR has also been observed regarding the inhibition of psoriatic inflammation, which only emphasizes the versatile properties of luteolin which decrease inflammatory skin reactions [144]. 

On a molecular basis, luteolin and, more significantly, methoxyluteolin have been found to inhibit the IgE-mediated release of histamine, leukotriene, prostaglandin D2, and GM-CSF from mast cells, which play a significant role in ACD [154,155]. Moreover, methoxyluteolin can also inhibit mTOR activation in mast cells [156], consequently preventing the differentiation of naïve T cells into Th1 and Th17 cells involved in ACD [157]. The inhibition of mTOR activation leads to a block of neuropeptide-mediated TNF-α, IL-8, and VEGF release, preventing undesirable immune response progression [158]. Furthermore, mast cell activation was observed by Kempuraj et al. during pretreatment with luteolin [149], while Góngora et al., after using a methanol extract containing luteolin 7-O-β-glucoside, discovered an ACD inhibition rate of 49% at 24 h and 79% at 96 h [159]. Additional positive effects of luteolin include its suppression of hyaluronidases, thus inhibiting the breakdown of anti-inflammatory high-molecular-weight HA into proinflammatory low-molecular-weight HA and suppressing the release of proinflammatory cytokines by keratinocytes and fibroblasts [152]. This flavonoid has been found to promote the expression of anti-inflammatory cytokines such as IL-10 and regulatory T cells, which was observed in an asthma model [156]. Luteolin has been found effective in other forms of dermatitis, like atopic dermatitis (AD) [160]. Jo et al. used the extract from *Stellera chamaejasme* (L.) of which luteolin 7-O-glucoside was the major component in murine AD models. Consequently, decreased serum levels of IgE and IL-4 were observed with diminished epidermal thickening, lower transepidermal water loss, and increased skin hydration, which suggested strong antiatopic activity of the used flavonoid [160]. Jegal et al. established that luteolin 7-methyl ether, a compound isolated from the methanol extract of *W. ganpi* might present potential in AD treatment [161]. Flavonoids have been shown to have anti-inflammatory and antiallergic properties, and since flavonoids and coumarins are key components of *W. ganpi*, it was proposed that the antiatopic action of this plant’s extract is most likely generated by flavonoids [148,161]. In RBL-2H3—a rat basophilic leukemia cell line used to assess allergic reactions—luteolin 7-methyl ether significantly inhibited the PI-induced IL-4 mRNA upregulation which is strongly correlated with AD [161]. Additionally, the antiatopic activity of the flavonoid was tested in HaCaT cells treated with TNF-α [161]. In these cells, luteolin 7-methyl ether presented the ability to reduce the expression of IL-6 which TNF-α normally stimulates. Furthermore, pretreatment with luteolin 7-methyl ether decreased the TNF-induced expression of G-CSF and GM-CSF that are present in keratinocytes of AD patients. Interestingly, itching that remains still a significant nuisance in atopic dermatitis could be potentially an aim for luteolin, as luteolin 7-methyl ether significantly decreased the TNF-α-induced TRPV1 expression and slightly diminished the expression of TRPA1 and IL-31 induced by the same cytokine as well [161]. Therefore, luteolin 7-methyl ether can potentially be a therapeutic agent for AD.

Apart from this, in a house dust mite (HDM)-driven murine Balb/c model of allergic rhinitis, luteolin, the active component of *Perilla frutescens* (L.), was demonstrated to block the secretion of IL-4. This was also observed in mononuclear cells isolated from the peripheral blood of allergic rhinitis patients who had been restimulated with HDM [162]. 

## 15. Diabetes Mellitus

**Figure 11:**
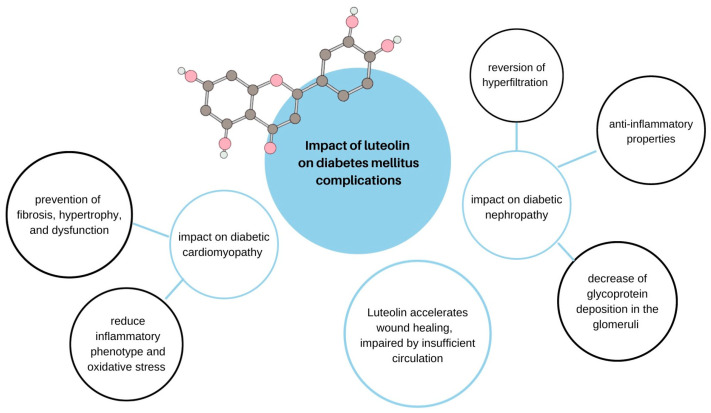
Schematic visualization of the impact of luteolin on treatment of diabetes mellitus complications [186,187,188].

Diabetes mellitus is a chronic metabolic disorder in which an organism cannot maintain the right glucose serum level due to the body’s inability to produce or use insulin effectively. According to WHO, it affects 422 million people worldwide, and its prevalence has been steadily increasing. Numerous complications of diabetes have been described, e.g., retinopathy, kidney failure, diabetic foot, cardiovascular disease, and overall decreased quality of life, which are consequences of two mechanisms: macro/microvascular angiopathy and neuropathy [163,164]. Studies have shown that oxidative stress and inflammation play an important role in the pathogenesis of diabetes mellitus [165,166]. Obesity is defined as BMI above 30kg/m^2^, though it is primarily the dysregulation of fat tissue, in which high endocrine activity combined with the production of reactive oxygen species (ROS) is a known cause of diabetes; however, the relationship between diabetes and obesity is the chicken or the egg causality dilemma [167,168]. The priority of managing diabetes ought to be regular physical activity, which contributes to the development of muscular tissue and weight management; also, it reduces the risk of diabetes [169,170]. The glucose serum level in muscles can be effectively lowered through the GLUT4 receptor, and there are numerous pathways by which exercise can increase the GLUT4 expression in skeletal muscle [171]. However, it is not sufficient in some patients, and others often do not maintain an appropriate level of physical activity, so pharmacotherapy must be prescribed. In recent years, many novel drugs were introduced to diabetes therapy: sodium–glucose co-transporter-2 (SGLT2), DPP-4 inhibitors (gliptins), and glucagon-like peptide-1 (GLP-1) analogs [172,173,174]. These drugs are very effective. However, their prices are high; thus, new compounds are being investigated, and one of them is luteolin. The pathogenesis of diabetes mellitus is multifactorial and involves various molecular pathways, including oxidative stress and inflammation [175,176]. Oxidative stress is a complex concept, and its definition has evolved to “An imbalance between oxidants and antioxidants in favor of the oxidants, leading to a disruption of redox signaling and control and/or molecular damage” [177,178]. Hyperglycemia leads to increased production of ROS, which may cause cellular damage and impact insulin action [179]. In the development of diabetes, inflammation is the second important factor [176]. Chronic low-grade inflammation is observed in type 1 and 2 diabetes and is associated with insulin resistance and beta-cell dysfunction [180]. A meta-analysis performed by Y. Wu et al. included five luteolin-related articles. The results showed that luteolin has a hypoglycemic effect in rat diabetic models, highlighting its potential use in clinical medicine; however, they implied that further clinical studies are essential [181]. Luteolin can also have an impact on diabetic conditions through its influence on complications. The anti-inflammatory, and antioxidant properties of luteolin significantly reduce oxidative and inflammatory damage in treated rats. Combined with the neuroprotective potential, it is a promising candidate for diabetes-induced lens neurodegeneration [166]. Another meta-analysis focused on different flavonoids (including luteolin) proved that luteolin plays a role in preventing type 2 diabetes [182]. Currently, there are no clinical trials involving luteolin and diabetes. According to the available PubMed data, two articles have been published since 2015, both describing the impact of nutraceutical Altilix^®^ (containing luteolin and chlorogenic acid extracts) on some biochemical parameters. The first one involved people with metabolic syndrome. After 6 months of supplementation with Altilix^®^, glycemic variables (HbA1c, HOMA-IR, and HOMA-β) had statistically significant improvements [183]. Other relevant parameters in diabetes mellitus pathogenesis and management—body weight and BMI, waist circumference, lipid profile (TC, TG, and LDL-C), hepatic enzymes (AST, ALT, AST/ALT)—also improvement. The same team from Italy in 2023 published their second article about Altilix^®^. This time they decided to look into the impact of Altilix^®^ on people from the original cohort with the BMI ≥ 25 and <30 kg/m^2^. Again, after six months, there was significant improvement in such parameters as body weight, glycemic, lipid parameters, but there was no improvement in waist circumference and HDL-cholesterol [184]. These results showcased that Altilix^®^ is effective in both patients with metabolic syndrome and patients at risk of developing it. What is important is that it affects glucose parameters. Luteolin exhibits its glucose-decreasing properties through the inhibition of α-glucosidase and α-amylase [185]. Several studies were performed on animals. One such study showed other interesting activity of luteolin. It accelerated wound healing, the process which is impaired in diabetic individuals because of insufficient circulation [186]. L. Chen et al. aimed to explore the luteolin’s impact on diabetic nephropathy [187]. They used 30 male Wistar rats, 20 of which received streptozotocin to induce hyperglycemia. The blood glucose level was significantly lower in the group receiving streptozotocin and luteolin. The impact on kidneys was evaluated by the level of creatinine, BUN (blood urea nitrogen), and electrolytes (sodium, potassium, chloride, calcium, phosphorus). Both creatinine and BUN, which are kidney function markers, were significantly decreased in the luteolin group. Diabetic kidney disease is a result of hyperfiltration, inflammation, and glycoprotein accumulation leading to fibrosis. Their results indicated that the reversion of hyperfiltration by luteolin, combined with its anti-inflammatory properties and the ability to decrease glycoprotein deposition in the glomeruli, can make it a very effective substance in the treatment of diabetic kidney disease. Another animal study involved C57BL/6 mice which were also injected with streptozotocin to induce diabetic cardiomyopathy, and high glucose was used to induce H9C2 cells injury in vitro [188]. Luteolin was able to prevent fibrosis, hypertrophy, and dysfunction in diabetic model mice. The experiment on H9C2 cardiomyocytes revealed luteolin’s capability to reduce inflammatory phenotype and oxidative stress (Figure 11). It is a result of altering two pathways: the inhibition of nuclear factor-kappa B (NF-κB) and the activation of antioxidant nuclear factor erythroid 2-related factor 2 (Nrf2) [188].

Some Chinese plant medicines exhibit good performance in controlling diabetes through reducing blood glucose levels and improving insulin resistance, possibly due to the presence of luteolin, yet their compositions are usually multicomplex; thus, medical intervention should not rely on such medicaments [189].

**Figure 12:**
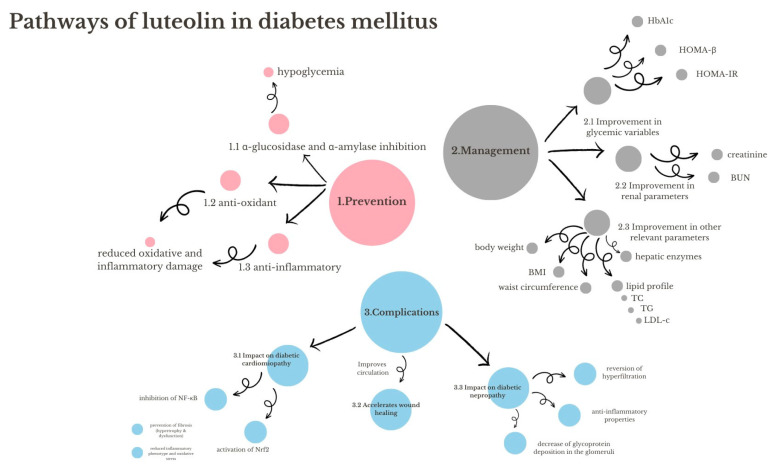
Schematic visualization of pathways that connect diabetes mellitus and luteolin [181,185,188,190].

Luteolin, like other flavonoids, influences glucose metabolism and its consequences when impaired (Figure 12). Studies on animals and humans highlight solid data about the positive impact of luteolin on diabetes and the associated metabolic conditions, yet more clinical trials focused on pure luteolin activity in the field of diabetes are necessary.

## 16. COVID-19

**Figure 13:**
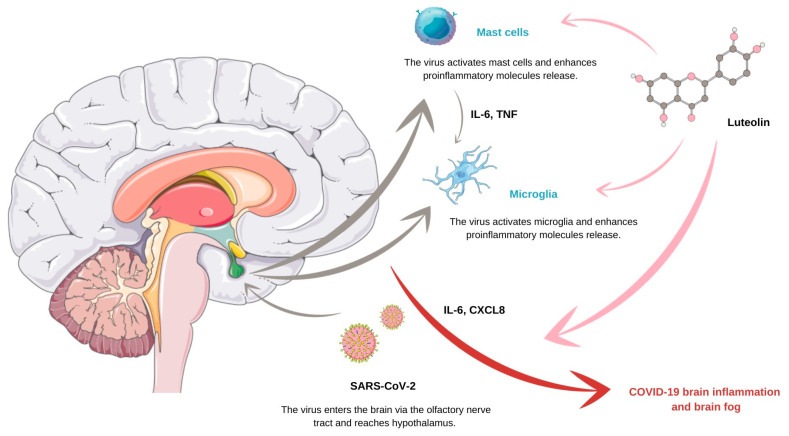
Schematic visualization of the ways in which SARS-CoV-2 affects mast cells and microglia as well as the impact of luteolin on this process [29,155,158,197,198,201].

When describing the connection between SARS-CoV-2 and luteolin, it is important to underline that the discussed flavonoid presents both neuroprotective and antiviral effects [10,191]. Neuroinflammation, which can be caused by traumatic brain injury (TBI), has consequences in neuroimmune cell activation, which leads to progressing neurodegeneration and cognitive defects [192]. Neurodegenerative diseases threaten the world of medicine with their invulnerability, as the current state of medical knowledge cannot recommend promising therapeutical options. Therefore, the neuroprotective potential of luteolin, which was proven to suppress neuroinflammatory responses in SARS-CoV-2, might be significant in managing neurodegenerative conditions [192]. The neuroprotective potential of the flavonoid might alleviate the severity of diseases such as multiple sclerosis, Parkinson’s disease, and Alzheimer’s disease, improving cognitive decline [193]. The antiviral effect of flavonoids was first suggested in 1990, when the infectivity of human and bovine coronaviruses, NCDCV and OC43, was reduced by 50% by quercetin in concentrations ranging from 60 μg/mL [194]. When it comes to the specific antiviral effect on SARS-CoV-2, recent research hypothesizes that flavonoids can inhibit transmembrane peptidase serine 2 (TMPRSS2) as well as endo-protease Furin, which is responsible for SARS-CoV-2 Spike protein cleavage and therefore for facilitating its infectivity [195]. Therefore, by inhibiting the described proteases, flavonoids might be able to suppress the propagation of the viral infection. Luteolin can prevent brain inflammation and brain fog connected with the COVID-19 disease course [196]. SARS-CoV-2 can enter the brain via the olfactory nerve tract, and in this way, the virus reaches the hypothalamus, which enhances the proinflammatory molecules released by brain mast cells and microglia [197,198]. The described process leads to brain inflammation and brain fog, which are exacerbated by neuropeptides released under stressful conditions as well as chemotherapy. Luteolin can inhibit both mast cells and microglia and reduce neuroinflammation and cognitive dysfunction (Figure 13). Its ability to suppress astrocytes and oxidative stress enhances its neuroprotective effects. Not to mention that the antiviral properties of the discussed flavonoid are wider, as it inhibits the entry of viruses into the host cell [158]. Nevertheless, the absorption of luteolin after oral administration, which has been already described, remains problematic [199]. For this reason, it is administered in liposomal preparations using olive pomace oil, which improves its bioavailability and provides additional anti-inflammatory and neuroprotective effects [200].

Recent research proves that luteolin therapy can increase the rate of recovery of smell after COVID-19 infection, which is of great importance as almost two-thirds of patients diagnosed with COVID-19 report hyposmia or anosmia, up to 20% of which present impaired sense of smell a long time after infection [191].

## 17. Conclusions

The application of substances of natural origin appears very effective, as the described compounds present considerably fewer side effects in therapy. Although the current state of knowledge provides us with many question marks, the anticancer potential of luteolin, a widespread organic compound, lets us presume that its use in the therapy of varied neoplasms will develop with increased treatment effectiveness in the future. 

In neoplastic diseases, luteolin influences various signaling and metabolic pathways; therefore, it presents the activity favored in treating these illnesses. The main attribute of this flavonoid is that it induces the apoptosis of cancer cells and lowers the chances of metastases. Additionally, in breast cancer, the nephrotoxic activity of cisplatin can be mitigated by the administration of luteolin, which, combined with its anticancer qualities, might be a good reason to use it in therapy with this chemotherapeutic. Another therapeutic possibility is observed in bone metastases of lung cancer, where serious pain felt by patients could be treated with doses of luteolin. The dualistic nature of the substance and the multitude of how it interferes with molecular pathways makes luteolin a very interesting research subject, and, hopefully, even more applications of the flavonoid will be discovered.

When it comes to nonneoplastic diseases, the fact that luteolin can improve treatment quality in dermatological and diabetic patients and prevent brain inflammation in coronaviral infections is undeniable. In coronaviral infections, luteolin’s qualities, being the suppression of neuroinflammatory responses, positive neuroprotective effect, and the inhibition of proteases facilitating infectivity, may be beneficial not only in reducing the infectivity of the virus but also in preventing severe complications such as brain fog and brain inflammation and additionally increasing the rate of smell recovery. Moreover, the impact of the described flavonoid on other neuroinflammatory conditions, such as multiple sclerosis and Alzheimer’s disease, lets us hope that the prevention of neurodegenerative diseases will improve in years to come. 

The beneficial influence of luteolin has been observed regarding diabetes mellitus. Since its hypoglycemic characteristics and a positive impact on laboratory parameters—biochemical, glucose, and lipid profiles—luteolin may be used in the course of time in diabetes mellitus—both in prophylaxis and therapy. Moreover, in rodents, this naturally derived substance presented promising results considering long-term complications—diabetic kidney disease, cardiomyopathy, and neurodegeneration. The described effects of luteolin in diabetes mellitus have been discovered in animals; therefore, more research is needed to establish whether similar correlations exist in humans. 

Dermatological patients are another group that may benefit from luteolin in their therapy. In murine psoriatic models, it has been observed that luteolin diminishes keratinocyte proliferation, resulting in decreased scale thickness. Moreover, it stimulates cortisol production, enhancing its anti-inflammatory properties. If similar outcomes could be found in humans, the quality of life of patients with psoriasis would significantly improve. In allergic contact dermatitis, luteolin in the form of lotion has presented qualities of diminishing redness and increasing skin hydration, therefore preventing irritation from frequent washing and irritants. Itching that remains an extensive challenge in treating atopic dermatitis could also be an aim for luteolin. This substance, when used in murine models, resulted in reduced IgE and IL-4 concentrations in serum, as well as reduced epidermal thickening and transepidermal water loss; therefore, it offers vast possibilities that could be discovered in patients, thus contributing to their improved life. 

Luteolin as a substance that shows remarkable promise of being a highly effective drug with a broad spectrum of applications. Studies on cell and rodent models deliver truly encouraging results accompanied by the safety of this drug. One of the problems associated with the possibility of using luteolin is the form of its administration. The oral path might not be effective as this flavonoid has poor absorption from the intestine, so different distribution methods have to be discovered to enable the use of this substance as a medication. A different challenge that emerges from various articles is that very little research is performed on patients. Rodent models are excellent at showing the capabilities of a substance; however, we cannot be sure of the real-life applications of luteolin until the research with humans has been conducted. In conclusion, two major pathways to work in future research connected with luteolin are discovering new forms of the administration of the drug and inspecting the effect this substance has on humans.
